# Knowledge, attitudes, and practices regarding cervical cancer and screening among women visiting primary health care Centres in Bahrain

**DOI:** 10.1186/s12889-018-5023-7

**Published:** 2018-01-11

**Authors:** Ghufran Jassim, Alaaeddin Obeid, Huda A. Al Nasheet

**Affiliations:** Family Medicine Department, Royal College of Surgeons in Ireland –Medical University of Bahrain, Busaiteen Bahrain, Box 15503, Adliya, PO Bahrain

**Keywords:** Cervical cancer, Women, Screening, Human papillomavirus (HPV) vaccine

## Abstract

**Background:**

Cervical cancer is one of the most common cancers among women, with 80% of the cases occurring in developing countries. Cervical cancer is largely preventable by effective screening programs. This has not been possible with opportunistic screening and its low use in the Kingdom of Bahrain. The objective of this study was to explore the knowledge, attitudes, and practices of women attending primary care health centres for cervical cancer screening.

**Methods:**

This was a cross-sectional study of 300 women attending primary health care centres in Bahrain. We used a validated tool comprised of 45 items to collect data through face-to-face interviews between December 2015 and February 2016. Descriptive data are presented for demographic data, and frequency distributions with percentages are presented for each item of the knowledge and attitude questionnaire.

**Results:**

The mean age ± SD of the participants was 37.24 ± 11.89 years, they were mostly married (221; 73.7%), and had a high school or higher education (261; 87%). Over 64% (194 participants) had never heard of a Pap smear procedure and only 3.7% (11 participants) had heard about the human papillomavirus (HPV) vaccine. Nearly 64% (192 participants) believed that a Pap smear was helpful in detecting pre-cancer and cancer of the cervix, and 44.3% (133 participants) believed that they should have a Pap smear at least every 3 years. Regarding the practice, only 40.7% (122 participants) had a Pap smear in their lifetime. The majority of participants felt embarrassed when examined by a male doctor (250, 83.3%) and few underwent a Pap smear screening if they were never married (69, 23.0%).

**Conclusions:**

The participants demonstrated a wide range of knowledge and attitudes towards cervical cancer screening. However, the majority demonstrated positive attitudes towards the HPV vaccine.

## Background

Cervical cancer is the fourth most frequently diagnosed cancer with an estimated 527,600 new cases in 2012 worldwide [1, 2]. It is the fourth leading cause of cancer death with 265,700 deaths among women worldwide in 2012 [1]. Nearly all cases of cervical cancers can be attributed to human papillomavirus (HPV) infections [3]. Over 120 types of HPV have been identified and 12 types (HPV 16, 18, 31, 33, 35, 39, 45, 51, 52, 56, 58, and 59) showed sufficient evidence to be causally linked with the development of cervical cancer [4].

Screening is the principal preventive measure used to reduce the burden of cervical cancer. The main purpose of cervical cancer screening is the identification of early-stage invasive cancer. This is achieved through use of the conventional cytology-based Papanicolaou smear (Pap smear) to identify cervical cancer precursors that can be removed before progression to invasive cancer [5].

Using the Pap smear test in population-based screening programs has helped reduce the incidence and mortality of cervical cancer by up to 80–90% in several developed countries over the last five decades [5, 6].

The HPV vaccination was first introduced in 2006, targeting females between the ages of 9–14 years with the aim of preventing several types of HPV that cause cervical precancerous lesions and cancer [3, 7].

In the Kingdom of Bahrain, cervical cancer is the fourth most frequent cancer with an approximate incidence rate of 4.3 per 100,000 individuals per year, with an age standardised incidence rate of 5.9. The cervical cancer incidence rate in Bahrain is the 2nd highest in the Gulf Corporation Council (GCC) countries after the UAE (ASR 9.5) [7].

In Bahrain, current technology has not translated into an effective national, population-based, cervical screening program*.* Cervical screening is still an opportunistic event, depending on women encountering their health care providers, with an estimated coverage of 43.1% [7]. The HPV vaccine is not yet available within free public health services; however, it can be obtained in private health institutions upon request.

When considering the significant impact of cervical screening, the low coverage of cervical screening, and absence of a population-based screening program, as well as not including the HPV vaccine in the national vaccination schedule, knowledge assessment of cervical cancer screening and the HPV vaccine is an important determinant for cervical cancer screening acceptance and use.

## Methods

### Study setting and population

In this cross-sectional study, we recruited 300 Bahraini females attending the primary health care centres. We adopted several stages for our sampling technique. First, all five health governorates were included as our sample strata. Then, one health centre was selected at random from all the health centres belonging to each health governorate. Finally, systematic sampling was adopted by selecting every 5th woman attending the health centre for any reason during December 2015 to February 2016. All governmental health care centres provided screening services free of charge for nationals.

### Ethics

Ethics approval was obtained from the Royal College of Surgeons in Ireland-Medical University in Bahrain and the Ministry of Health in the Kingdom of Bahrain. Written consent was obtained from participants before commencing the interviews.

### Sample size

A previous study [8] yielded a mean ± SD knowledge score of 59.4 ± 24.3. We set a 95% confidence interval within three scores of the population using the following:

$$ {\displaystyle \begin{array}{l}n={\left(\frac{z^{\ast }s}{ME}\right)}^2\\ {}={\left({1.96}^{\ast }24.3/3\right)}^2\\ {}=250\end{array}} $$

A total of 300 participants were enrolled in the study allowing for a 20% non-response rate. Data collection was done using a validated questionnaire that was adopted from a previous study [9]. The questionnaire consisted of 45 items addressing personal data as well as questions regarding knowledge, attitudes, and practices related to cervical cancer screening. The questionnaire was administered through face-to-face interviews by trained Arabic speaking female interviewers. Data were entered into the SPSS statistical package, version 22 (SPSS, Chicago, IL, USA).

### Data analysis

Descriptive data are presented for demographic data and frequency distribution with percentages presented for each item in the knowledge and attitude questionnaire (Table 3). Association was tested between the independent demographic variables and “Knowing about Pap smear test” using the chi-square test for dependent categorical variables (i.e., education status, employment, and marital status). The same method was used for Practice (“Have you ever had a Pap smear done?”) and Attitude (“Pap smear is unnecessary if there are no signs or symptoms”) data.

## Results

### Population

In this study, 300 Bahraini females consented to participate and complete the questionnaire. The mean ± SD age of the participants was 37.24 ± 11.89 years, with a mean parity of 2.31 ± 2.09 and mean duration of marriage of 14.9 ± 10.99 years. Most of the participants were married (221; 73.7%), with a high school or higher education (261; 87%), and approximately 41% were employed (123 participants) (Table 1).

**Table 1:** Socio-demographic data of the participants (*N* = 300)

Variable	No (%)	
Marital status		
• Married	221 (73.7)	
• Single	55 (18.3)	
• Divorced	14 (4.7)	
• Widow	10 (3.3)	
Educational status		
• Illiterate	14 (4.7)	
• Primary	10 (3.3)	
• Preparatory	15 (5.0)	
• Secondary	118 (39.3)	
• University	143 (47.7)	
Employment status		
• Employed	123 (41.0)	
• Unemployed	160 (53.3)	
• Retired	17 (5.7)	
Variable		Mean (SD)
Age		37.24 (11.89)
Parity		2.31 (2.09)
Duration of marriage		14.95 (10.99)

### Knowledge

Nearly 65% (194 participants) had heard about the Pap smear. The main source of information was from a gynaecologist (51.5%) followed by relatives and friends (18%), the media (13.4%), family physicians (12.4%), and nurses (3.6%).

Table 2 shows that approximately 64% (192 participants) believed that the Pap smear was helpful in detecting pre-cancer and cancer of the cervix, 44.3% (133 participants) believed that they should have a Pap smear at least every 3 years, and 67.7% (203 participants) knew that the purpose of the Pap smear was to detect abnormal cells in the cervix. Nevertheless, 10% (30 participants) believed that the Pap smear is not successful in reducing the incidence and mortality of cervical cancer.

**Table 2:** Knowledge and beliefs of the women about cervical cancer risk and prevention (*N* = 300)

Knowledge/belief item	Yes No. (%)	No No. (%)	Don’t know No. (%)
1. Have you ever heard about the Pap smear?	194 (64.7)	106 (35.3)	–
2. Family history of cervical caner	20 (6.7)	280 (85.3)	–
3. Women should have Pap Smears at least every 3 years	133 (44.3)	19 (6.3)	148 (49.3)
4. Pap smear is the most helpful way to detect pre-cancer and cancer of the cervix	192 (64.0)	2 (2.0)	102 (34.0)
5. Pap smear is not able to detect precancerous cells before manifestations of its symptoms	49 (16.3)	70 (23.3)	181 (60.3)
6. The purpose of the Pap smear is to detect abnormal cells in the cervix	203 (67.7)	4 (1.3)	93 (31.0)
7. Pap smear is not successful in reducing incidence and mortality of cervical cancer	30 (10.0)	107 (35.7)	163 (54.3)
8. Pap smear is able to detect all types of female genital cancer	119 (39.7)	31 (10.3)	150 (50.0)
9. Pap smear is a non-invasive and relatively inexpensive method	117 (59.0)	9 (3.0)	114 (38.0)
10. Women should have Pap smear since the onset of sexual activity	101 (33.7)	38 (12.7)	161 (53.7)
11. In Pap smear, cervical cells are examined	154 (51.3)	10 (3.3)	136 (45.3)
12. Pap smears can be performed at both menstrual and non- menstrual period	35 (11.6)	105 (34.7)	163 (53.8)
13. A woman should not have sex 24 h before having Pap smear	103 (34.3)	21 (7.0)	176 (58.7)
14. Pap smear should be discontinued after menopause	26 (8.7)	121 (40.3)	153 (51.0)
15. If someone is having a normal Pap smear, she does not need Pap smears in the future	39 (13.0)	124 (41.3)	137 (45.7)
16. There is no need to have a Pap smear if it is not administered by a doctor	17 (5.7)	213 (71.0)	70 (23.3)
17. Have you heard about HPV vaccine?	11 (3.7)	190 (63.3)	99 (33.0)

Approximately 59% of the respondents (117 participants) believed that the Pap smear is non-invasive. Approximately 33.7% (101 participants) thought that women should have Pap smears from the onset of their sexual activity, and 34.3% (103 participants) thought that Pap smears could not be performed during menstrual periods and agreed that women should not have sex 24 h before having a Pap smear. Only 8.7% (26 participants) believed that a Pap smear should be discontinued after menopause. Finally, 71% (213 participants) agreed to having a Pap smear even if not performed by a doctor. Regarding the HPV vaccine, only 3.7% (11 participants) had heard about the vaccine, and the majority (289; 96.3%) either had not heard or did not know about the HPV vaccine.

### Attitudes and practices

Regarding Pap smear practice, only 40.7% (122 participants) had undergone a Pap smear in their lifetime, 45.5% preferred to have it done at a gynaecology clinics, and 16.4% preferred to have a Pap smear done in a Primary Health Care Centre.

Table 3 shows the responses to each item in our attitude and practice questionnaire. The majority (250; 83.3%) felt embarrassed if a male doctor performed the test and only 23.0% (69 participants) would go for screening if they were unmarried.

**Table 3:** Attitudes and practices of women towards cervical cancer screening (*N* = 300)

Attitude/Practice item	Yes No. (%)	No No. (%)	Don’t know No. (%)
1. Have you ever had a Pap smear done?	1221 (40.7)	155 (51.7)	23 (7.7)
2. If male doctor performed the test, would you feel embarrassed?	250 (83.3)	42 (14.0)	8 (2.7)
3. If you were single, would you go for screening test?	69 (23.0)	173 (57.7)	58 (19.3)
4. Are you discouraged from screening for cervical cancer by your partner or others?	28 (9.3)	208 (69.3)	64 (21.3)
5. Do you have a fatalistic attitude?	146 (48.7)	114 (38)	40 (13.3)
6. Is it painful to have a Pap smear?	58 (19.3)	76 (25.3)	166 (55.3)
7. Having a Pap smear is unpleasant/embarrassing	107 (35.7)	67 (22.3)	126 (42.0)
8. It is difficult to take time off to go for Pap smear	53 (17.7)	169 (56.3)	78 (26.0)
9. It is difficult to get to the Pap smear clinic	40 (13.3)	197 (65.7)	63 (21.0)
10. Being busy is a barrier to Pap smear	77 (25.7)	179 (59.7)	44 (14.7)
11. Pap smear is unnecessary if there are no signs and symptoms	59 (19.7)	188 (62.7)	53 (17.7)
12. Is it unnecessary to go only for Pap smear test	59 (19.7)	186 (62.0)	55 (18.3)
13. Going for Pap smear screening is too expensive	22 (7.3)	111 (37.0)	167 (55.7)
14. I am afraid that something wrong will be detected if I go for Pap smear	79 (26.3)	150 (50.0)	71 (23.7)
15. I am uneasy about talking about cancer	165 (55.0)	117 (39.0)	18 (6.0)
16. I would be worried if I was found to have early signs of cancer	217 (72.3)	57 (19.0)	26 (8.7)
17. Would you take HPV vaccine?	245 (81.8)	55 (18.2)	–
18. Would you allow your children to be vaccinated against HPV?	273 (90.9)	27 (9.1)	–

Only 9.3% (28 participants) were discouraged by their partner or others from undergoing a Pap smear screening for cervical cancer. Nearly half of participants (146; 48.7%) had a fatalistic attitude, and 35.7% (107 participants) felt that the Pap smear procedure was unpleasant or embarrassing, while 19.3% (58 participants) thought it was painful.

Approximately 55% (165 participants) of the women in this study were uneasy when talking about cancer and 72.3% (217 participants) would be very worried if they were diagnosed with early signs of cancer.

Regarding the HPV vaccine, 81.8% (245 participants) would be vaccinated, and 90.9% (273 participants) would allow their children to be vaccinated against HPV.

Tests of associations (Table 4) showed that married women, knowledge concerning the Pap smear (chi-square = 45.943; *p* < 0.001), use of the Pap smear (chi-square = 52.883; *p* < 0.001), and positive attitudes towards the Pap smear (chi-square = 14.623; *p* = 0.023) were significantly more associated than their counterparts. Educational status reached significance only with attitude, whereby more educated women believed that a Pap smear was necessary even in the absence of signs and symptoms (chi-square = 15.718; *p* = 0.047).

**Table 4:** The association between knowledge, attitude, and practice and the independent socio-demographic variables

Variable	Pearson chi square	*p*-value	df^a^
Knowledge: Have you heard of the Pap smear			
Marital status	45.943	0.000	3
Educational level	3.325	0.505	4
Employment	2.629	0.269	2
Practice: Have you ever had a Pap smear done			
Marital status	52.883	0.000	3
Educational level	6.738	0.565	4
Employment	3.132	0.536	2
Attitude: A Pap smear is unnecessary if the patient has no signs or symptoms			
Marital status	14.623	0.023	3
Educational level	15.718	0.047	4
Employment	9.153	0.057	2

^a^Degree of freedom

## Discussion

This was a cross-sectional study of 300 women to determine their knowledge, attitudes, and practices towards cervical cancer. The results showed that most women (65%) had heard about the Pap smear and understood its importance in detecting precancerous lesions and reducing the incidence and mortality of cervical cancer. However, only 40% of the women had underwent a Pap smear in their lifetime. Similar percentages of women undergoing the Pap smear have been reported in previous studies conducted in other Arab countries such as the United Arab of Emirates and Qatar [9, 10]. This level of uptake and knowledge is far less than that reported in developed countries, although the data were at least a decade older [11].

The percentage of participants responding correctly to questions regarding the specifics and requirements of the procedure, such as when to start the screening, the best time of the menstrual cycle to have it done, not having sex 24 h prior to the procedure, and not to discontinue having a Pap smear after menopause ranged between 11 and 34%, reflecting suboptimal knowledge in this study.

Considering the religious and conservative aspects of this culture, the majority of participants expressed their feeling of embarrassment if examined by a male doctor, and had been denied screening if single. Few participants were discouraged from cervical screening by their partner or friends, however, this study failed to determine the reasons for this finding.

In contrast to previously published studies that showed a linear relationship between education and knowledge of cervical cancer, in this study, knowing about a Pap smear was not influenced by education or employment. However, these findings should be interpreted cautiously because the association was not based on a total score of all knowledge items, but was based on only one item.

Regarding the HPV vaccine, although only 3.7% of the participants had ever heard of the vaccine, the majority reported positive attitudes towards it and were happy to either take the vaccine themselves or allow their children to be vaccinated. Similar high acceptance rates of the HPV vaccine were reported in another study conducted in the Kingdom of Bahrain [12]. This information should encourage health policy officials to promote the HPV vaccine as it resolves the dispute presented in previous studies about parental perception of the HPV vaccine encouraging premarital sexual behaviour [13, 14].

Studies in other developing countries such as India, Nepal, Ethiopia, and Nigeria reported a lower level of knowledge regarding cervical cancer but favourable attitudes towards screening. The low level of knowledge was attributed in many instances to a lack of population-based screening programs, inefficient mass media campaigns, or cultural barriers [15–17].

This study had some limitations. The results could not be generalized to the entire population of Bahrain because our sample was obtained from primary health care centres that provide free health care services to Bahrain nationals. There were a few private practices that were not included in the study. One should be cautious when interpreting the association between knowledge, practices, attitudes, and independent socio-demographic variables because this association was not based on a total score of all items. Instead, it was based on one item. Additionally, we did not explore the reasons/barriers for low use of the Pap smear, which could constitute a basis for future research. Nevertheless, the study had some strengths, including the use of a probability multi-stage sampling technique, high response rate, and use of a standardised tool to assess the outcomes. Additionally, this survey provided baseline information for future planning of cervical cancer prevention and screening programs in the Kingdom of Bahrain.

## Conclusions

Participants demonstrated a wide range of knowledge and attitudes towards cervical cancer screening. However, the majority demonstrated a positive attitude towards the HPV vaccine. This study shows the need to establish a sustainable awareness campaign concerning the prevention of cervical cancer, and further emphasizes the importance of a nationwide population-based screening program across primary health care centres.

## References

[1] Cervical Cancer: Estimated incidence, mortality and prevalence worldwide in 2012 [http://globocan.iarc.fr/old/FactSheets/cancers/cervix-new.asp]. Accessed 25 Nov 2015.

[2] GLOBAL BURDEN OF CANCER IN WOMEN. Current status, trends, and interventions [https://www.cancer.org/content/dam/cancer-org/research/cancer-facts-and-statistics/global-cancer-facts-and-figures/global-burden-of-cancer-in-women.pdf]. Accessed 25 Nov 2015.

[3] Human papillomavirus (HPV) and cervical cancer [http://www.who.int/mediacentre/factsheets/fs380/en/]. Accessed 25 Nov 2015.

[4] Burd EM (2003). Human papillomavirus and cervical cancer. Clin Microbiol Rev.

[5] Denny L, Herrero R, Levin C, Kim JJ. Cervical Cancer. In: Cancer: Disease Control Priorities, Third Edition (Volume 3). edn. Edited by Gelband H, Jha P, Sankaranarayanan R, Horton S. Washington (DC): World Bank; 2015. 26913318

[6] Sankaranarayanan R (2014). Screening for cancer in low- and middle-income countries. Ann Glob Health.

[7] Bruni L, Diaz M, Barrionuevo-Rosas L, Herrero R, Bray F, Bosch FX, de Sanjose S, Castellsague X (2016). Global estimates of human papillomavirus vaccination coverage by region and income level: a pooled analysis. Lancet Glob Health.

[8] Sedighe Rezaie-Chamani SM-A-C, Kamalifard M (2012). Knowledge, attitudes and practice about pap smear among women Reffering to a public hospital. Journal of Family and Reproductive Health.

[9] Al-Meer FM, Aseel MT, Al-Khalaf J, Al-Kuwari MG, Ismail MF (2011). Knowledge, attitude and practices regarding cervical cancer and screening among women visiting primary health care in Qatar. East Mediterr Health J.

[10] Metwali Z, Al Kindi F, Shanbleh S, Al Akshar S, Sarhan F (2015). Evaluating awareness and screening of cervical cancer among women in Sharjah, United Arab Emirates. IOSR Journal Of Pharmacy.

[11] CK Y, Rymer J (1998). Women's attitudes to and awareness of smear testing and cervical cancer. Br J Fam Plann.

[12] Moosa K, Alsayyad AS, Quint W, Gopala K, DeAntonio R (2014). An epidemiological study assessing the prevalence of human papillomavirus types in women in the Kingdom of Bahrain. BMC Cancer.

[13] Siu JY-m: Perceptions of and barriers to vaccinating daughters against human papillomavirus (HPV) among mothers in Hong Kong. BMC Womens Health 2014, 14:73–73. 10.1186/1472-6874-14-73 PMC4049476 24890226

[14] Davis K, Dickman ED, Ferris D, Dias JK (2004). Human papillomavirus vaccine acceptability among parents of 10- to 15-year-old adolescents. J Low Genit Tract Dis.

[15] Bansal AB, Pakhare AP, Kapoor N, Mehrotra R, Kokane AM (2015). Knowledge, attitude, and practices related to cervical cancer among adult women: a hospital-based cross-sectional study. J Nat Sci Biol Med.

[16] Nwankwo KC, Aniebue UU, Aguwa EN, Anarado AN, Agunwah E (2011). Knowledge attitudes and practices of cervical cancer screening among urban and rural Nigerian women: a call for education and mass screening. Eur J Cancer Care (Engl).

[17] Shrestha J, Saha R, Tripathi N: Knowledge, Attitude and Practice regarding Cervical Cancer Screening Amongst Women visiting Tertiary Centre in Kathmandu, Nepal. 2013 2013, 2(2):6.

